# Interplay between epigenetic alterations and piRNA expression in cancer development

**DOI:** 10.1080/15592294.2026.2758456

**Published:** 2026-11-09

**Authors:** Siyun Peng, Hangyu Wang, Ermao Li

**Affiliations:** Institute of Translational Medicine, Hengyang Medical College, University of South China, Hengyang, Hunan, China

**Keywords:** piRNA, PIWI, epigenetics, epitranscriptomics, DNA methylation, histone modifications, cancer

## Abstract

PIWI-interacting RNAs (piRNAs) are small non-coding RNAs generated from longer precursor transcripts through primary processing and, in selected biological contexts, a secondary ping-pong amplification cycle. They associate with PIWI proteins to target and silence transposable elements (TEs), and dysregulation of the PIWI-piRNA pathway has been reported in multiple human cancers. In physiological germline models, epigenetic modifications – including DNA methylation, histone modifications, RNA modifications, and chromatin remodeling – coordinately regulate the activity of piRNA clusters to ensure proper piRNA biogenesis. However, in tumor cells, how epigenetic modifications influence piRNA transcription and processing remains poorly characterized, with most studies focusing on how dysregulated piRNAs drive tumorigenesis via epigenetic or epitranscriptomic mechanisms. This review also discusses species-specific differences, limitations in somatic piRNA annotation, and the evidence needed to distinguish association from mechanism. DNMT/HDAC inhibitors, dietary bioactive compounds, and deactivated Cas9 (dCas9)-based epigenome editing may provide useful experimental or therapeutic approaches, but piRNA-directed epigenetic therapy remains preclinical.

## Introduction

### Epigenetic and epitranscriptomic regulation

Epigenetic regulation refers to changes in gene activity and chromatin state that occur without altering the underlying DNA sequence. DNA methylation can influence transcription through covalent modification of cytosines, whereas histone modifications such as methylation and acetylation can alter chromatin accessibility. Non-coding RNAs provide an additional regulatory layer by interacting with nucleic acids or proteins to influence transcriptional and post-transcriptional processes. Major mechanisms include DNA methylation, histone modifications, and chromatin remodeling [1,2]. RNA modifications act directly on RNA molecules and are therefore described here as epitranscriptomic regulation. These concepts are introduced before piRNA biology because the central aim of this review is to discuss their bidirectional relationship with the PIWI-piRNA pathway in cancer.

### Nature and functions of piRNAs

Argonaute (AGO) proteins are central components of small-RNA silencing pathways and commonly bind miRNAs or siRNAs to regulate target RNAs. PIWI proteins constitute a specialized Argonaute subfamily that associates predominantly with piRNAs [3]. PIWI-interacting RNAs (piRNAs) are a class of small non-coding RNAs that specifically interact with PIWI proteins. They are typically 23–30 nucleotides (nt) in *Drosophila melanogaster* and 24–31 nt in mammals; similar size ranges have been reported in somatic tissues, although annotation outside the germline requires caution [3,4]. They bear a conserved 2’-O-methylation modification at their 3’ termini, which significantly enhances their molecular stability. piRNAs were first identified in *D. melanogaster* and mammalian germ cells, are predominantly transcribed from discrete piRNA clusters in the genome, and primarily function to maintain genomic integrity by silencing transposable elements (TEs), as well as participate in germ cell development and stem cell homeostasis [5,6]. However, accumulating evidence in recent years has revealed that piRNAs are also aberrantly expressed in somatic cells and are closely implicated in the pathogenesis of various diseases, especially cancer, while the precise underlying molecular mechanisms remain under intensive investigation [3,4,7].

#### Evolution and species specificity

Genes in the piRNA pathway are evolutionarily tailored for transposon defense. piRNAs guide PIWI proteins to recognize and silence continuously evolving transposable elements, thereby safeguarding genomic integrity. This ongoing evolutionary arms race between host defense mechanisms and invading genetic elements drives the remarkable diversity of the piRNA pathway across different species, leading to the hypothesis that piRNA pathway genes evolve more rapidly than ordinary genes. This hypothesis has been further validated by studies demonstrating that several piRNA-related genes are indeed rapidly evolving [8–10]. The piRNA silencing machinery has undergone rapid co-evolution in a species-specific manner. Li et al. discovered that the nucleic acid-binding channels of *Mus musculus* MILI (PIWIL2) and human HILI/PIWIL2-containing piRISCs are wider than those of invertebrates [9]. The open seed gate of mammalian piRISCs confers greater tolerance to base mismatches in the piRNA seed region, enabling more efficient target binding and cleavage. This ensures precise regulation of transposable elements, prevents off-targeting of mRNAs and long non-coding RNAs (lncRNAs), modulates the expression of a subset of mRNAs required for spermatogenesis, and fulfills its role in protecting the mammalian germline genome [9]. Konstantinidou et al. generated a comprehensive roadmap of piRNA clusters across seven species. Their cross-species comparison of piRNA transcriptomic and genomic features highlights both similarities and variations, laying a foundation for deeper understanding of the conserved principles and species-specific variations in piRNA biology [11] (Figure 1D). *D. melanogaster* uses Piwi, Aubergine (Aub), and Ago3, mice encode PIWIL1 (MIWI), PIWIL2 (MILI), and PIWIL4 (MIWI2), whereas humans encode PIWIL1, PIWIL2, PIWIL3, and PIWIL4 [6,11,12]. These proteins and their functions should therefore be interpreted together with species information. Ping-pong amplification also differs among species: *D. melanogaster* uses reciprocal Aub-Ago3 cleavage, whereas mouse fetal male germ cells use MILI (PIWIL2) and MIWI2 (PIWIL4) with distinct roles in piRNA biogenesis and DNA methylation [13–15]. An equivalent pathway in human somatic or cancer cells remains insufficiently established [16,17].

**Figure 1.**
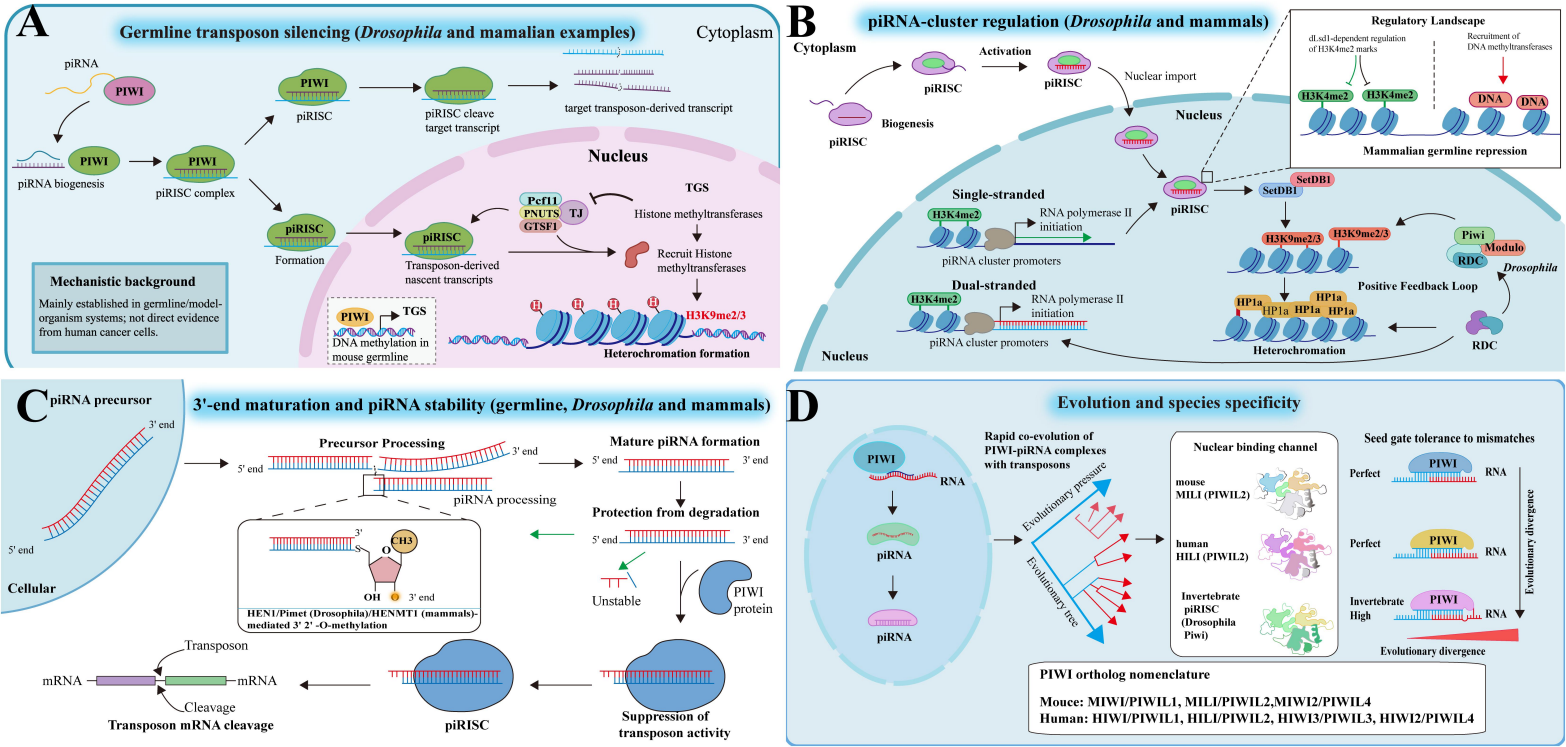
**A**: Germline transposon silencing (*D. melanogaster* and mammalian examples). piRNAs associate with PIWI proteins to form piRNA-induced silencing complexes (piRISCs), which repress transposons through post-transcriptional silencing in the cytoplasm and transcriptional silencing in the nucleus via heterochromatin formation. The mechanistic evidence shown here is derived mainly from germline and model-organism studies. **B**: piRNA-cluster regulation (*D. melanogaster* and mammals). Cytoplasmic piRISCs translocate into the nucleus and contribute to regulation of single-stranded or dual-stranded piRNA clusters through histone modifications and heterochromatin-associated factors. The inset summarizes a representative mammalian germline repression model. **C**: 3′-end maturation and piRNA stability (germline, *D. melanogaster* and mammals). Precursor processing, 3′ trimming, and HEN1/Pimet- or HENMT1-mediated 3′-end 2′-O-methylation generate stable mature piRNAs that load into PIWI proteins and support transposon silencing. **D**: evolution and species specificity. Rapid co-evolution between PIWI-piRNA pathways and transposons contributes to species-specific pathway features, including differences in PIWI orthologs and small-RNA recognition properties.

#### Transposon silencing

piRNAs play a critical role in safeguarding germline genome integrity from the deleterious effects of TEs [8]. Studies by Liu et al. have demonstrated that in the *D. melanogaster* germline, the transcription termination factor Pcf11 and phosphatase 1 nuclear targeting subunit (PNUTS) participate in piRNA-guided transposon silencing [18]. Pcf11 is recruited to nascent transcripts, which induces RNA polymerase II (Pol II) stalling, regulates Pol II elongation to halt transcription, and promotes proper gene transcription termination [18]. Similarly, Rivera et al. discovered in *D. melanogaster* that the transcription factor Traffic Jam (TJ) is a protein that associates with enhancer sequences of the piRNA locus Flamenco (Flam), and TJ suppresses transposons by activating Flam [19]. These mechanisms are best established in germline models; equivalent PIWI-piRNA pathways in human somatic or cancer cells require direct confirmation.

Upon binding to PIWI proteins, piRNAs assemble into the piRNA-induced silencing complex (piRISC). In *D. melanogaster*, this complex recognizes and binds to transposon-derived transcripts, thereby mediating gene silencing through two primary mechanisms: transcriptional gene silencing (TGS) and post-transcriptional gene silencing (PTGS). In the TGS pathway, silencing primarily relies on PIWI subfamily proteins with low catalytic activity. These proteins recruit histone-modifying enzymes, inducing alterations in chromatin structure and thus suppressing mRNA production at the transcriptional level. In mammals, piRNA pathways can additionally direct de novo DNA methylation of transposons [15,20]. In contrast, during PTGS, PIWI proteins directly cleave target transcripts to mediate their degradation at the post-transcriptional level [3]. Notably, *D. melanogaster* can still maintain efficient transposon silencing even under conditions of deficiency in certain piRNA biogenesis-related factors, indicating that this regulatory system possesses inherent compensatory or redundant mechanisms [4,5,7]. Gainetdinov et al. found that PIWI proteins exhibit greater tolerance to target mutations than AGO proteins and can efficiently cleave targets despite mismatches [21]. With the assistance of the cofactor gametocyte-specific factor 1 (GTSF1), PIWI proteins effectively cleave transcripts complementary to their bound piRNAs, protecting the genome from transposon invasion. This study confirms the evolutionary conservation of the piRNA pathway in transposon silencing [21]. Reuter et al. demonstrated in male mice that MIWI slicer activity directly cleaves LINE1 transcripts after embryonic DNA-methylation-based silencing has been established, providing post-transcriptional reinforcement independent of piRNA amplification [22]. By silencing transposons during their biogenesis and adapting specifically to target sequences, the piRNA pathway serves as a robust defense system that ensures genomic stability [9,23] (Figure 1A).

#### Chromatin remodeling and transcriptional regulation

In *D. melanogaster* germ cells, the piRNA pathway executes transposon defense through two distinct transcriptional modes, further establishing multi-layered epigenetic regulation at the chromatin level. H3K4me2 and H3K4me3 are generally associated with transcriptionally permissive or active chromatin. Single-stranded piRNA clusters bear specific H3K4me2 marks at their promoters, which permit Pol II to initiate transcription. The resulting piRISCs translocate back to the nucleus, recognize transcripts derived from piRNA clusters or nascent TE loci, and promote the establishment of H3K9me3, completing a self-reinforcing cycle that transitions chromatin from an ‘open’ to a ‘silenced’ state [24]. Double-stranded piRNA clusters are transcribed from both genomic strands and rely on the promoters of adjacent protein-coding genes for their transcription [25].

In *D. melanogaster*, although H3K9me3 is generally regarded as a repressive mark during canonical transcriptional activation and processing, during the transcription of piRNA clusters, Rhino – a homolog of Heterochromatin Protein 1 (HP1) – binds to H3K9me3 marks and recruits Deadlock and Cutoff to form the Rhino-Deadlock-Cutoff (RDC) complex, which activates transcription. These proteins act synergistically to ensure the smooth progression of transcription and transport the resulting piRNA precursor transcripts to the cytoplasm for processing [12]. After entering the nucleus, piRISCs execute the silencing program in a sequential manner. piRISCs recognize nascent TE transcripts, recruit the H3K9 methyltransferase SetDB1 to deposit H3K9me2/3, and subsequently recruit the heterochromatin protein 1a (HP1a) to form stable repressive chromatin. The *D. melanogaster* chromatin-associated protein Modulo interacts with Piwi to promote H3K9me3 enrichment at TE loci [26]. In addition, dLsd1 is required for Piwi-dependent TE silencing, and its depletion alters H3K4me2 and H3K9me2 at TE loci [27]. In the mouse male germline, piRNA pathways direct de novo DNA methylation of transposons through MIWI2 and downstream factors including SPOCD1 [15,20]. Thus, the piRNA pathway first initiates transcription of piRNA clusters via activating histone marks such as H3K4me2, and then through a cascade reaction involving piRISCs, chromatin-modifying enzymes, and silencing complexes, sequesters TEs within heterochromatin to achieve transgenerational epigenetic defense of the germline genome (Figure 1B). Comparable PIWI-directed chromatin mechanisms in human cancer remain less well established and are discussed in Bidirectional regulatory axis of epigenetics and piRNAs in cancer cells.

#### RNA modifications

Numerous distinct types of RNA modifications have been identified to date, including 5-methylcytidine (m5C), N^6^-methyladenosine (m6A), N^7^-methylguanosine (m7G), pseudouridine (Ψ), and N^1^-methyladenosine (m1A) [1]. Accumulating studies have demonstrated that small RNAs undergo diverse post-transcriptional modifications that modulate their functions in various biological processes [1,28]. Xiong et al. focused on 2’-O-methylation (Nm), a type of RNA modification in which a hydrogen (−H) atom on the 2’-hydroxyl (−OH) group of the ribose moiety is replaced by a methyl (-CH_3_) group [28]. Nm modification has been specifically detected at the 3’ termini of piRNAs. During piRNA biogenesis, HEN1 catalyzes the formation of Nm modification at piRNA 3’ ends, which confers a mature and stable structure to piRNAs and protects them from 3’-end degradation. These properly modified piRNAs subsequently maintain normal germ cell development and suppress TEs [28] (Figure 1C). In *D. melanogaster*, HEN1/Pimet methylates the 3’ termini of PIWI-associated piRNAs, whereas mammalian piRNAs are methylated by the homolog HENMT1. In mice, loss of HENMT1 reduces piRNA stability and length [28,29].

### Scope of this review

This article is a narrative review. It first summarizes the physiological and species-specific biology required to interpret the PIWI-piRNA pathway, and then focuses on evidence linking piRNAs with epigenetic or epitranscriptomic regulation in cancer. Findings from fruit fly, mouse, and other germline models are used as mechanistic background and are distinguished from mechanisms directly demonstrated in human cancer cells.

## Mechanisms of piRNA biogenesis under physiological conditions

The biogenesis mechanism of piRNAs is a complex and highly ordered regulatory process consisting of a primary processing pathway and, in selected biological contexts, a secondary ping-pong amplification cycle [30]. Transposon-enriched piRNA clusters in the genome are transcribed by Pol II into long single-stranded non-coding RNA precursors. These transcripts are transported to the cytoplasm and anchored to the outer mitochondrial membrane for further processing and modification. Endonucleases, including Zucchini (ZUC) in *D. melanogaster* and phospholipase D family member 6 (PLD6/MitoPLD) in mammals, cleave the 5’ ends of the long single-stranded RNAs, and the resulting intermediates are loaded onto PIWI proteins. 3’−5’ exonucleases (Nibbler and PNLDC1) then trim the intermediates to a length of 25–32 nucleotides, followed by HEN1 methyltransferase-catalyzed 2’-O-methylation at the 3’ termini, ultimately generating mature primary piRNAs. Primary piRNAs are amplified through the ping-pong amplification cycle. Primary piRNA-PIWI complexes (e.g., Aub in *D. melanogaster*) cleave complementary transposon transcripts to produce new intermediate RNA fragments. These fragments are loaded onto Ago3 proteins to form secondary piRNAs. Subsequently, secondary piRNAs cleave piRNA cluster transcripts to generate additional intermediate fragments, which are again loaded onto PIWI proteins to produce new primary piRNAs. These reciprocal cleavage steps form a self-sustaining ping-pong cycle that continuously amplifies the piRNA pool [13,31,32] (Figure 2). The ping-pong cycle is therefore not a universal or obligatory step in all piRNA biogenesis pathways.

**Figure 2.**
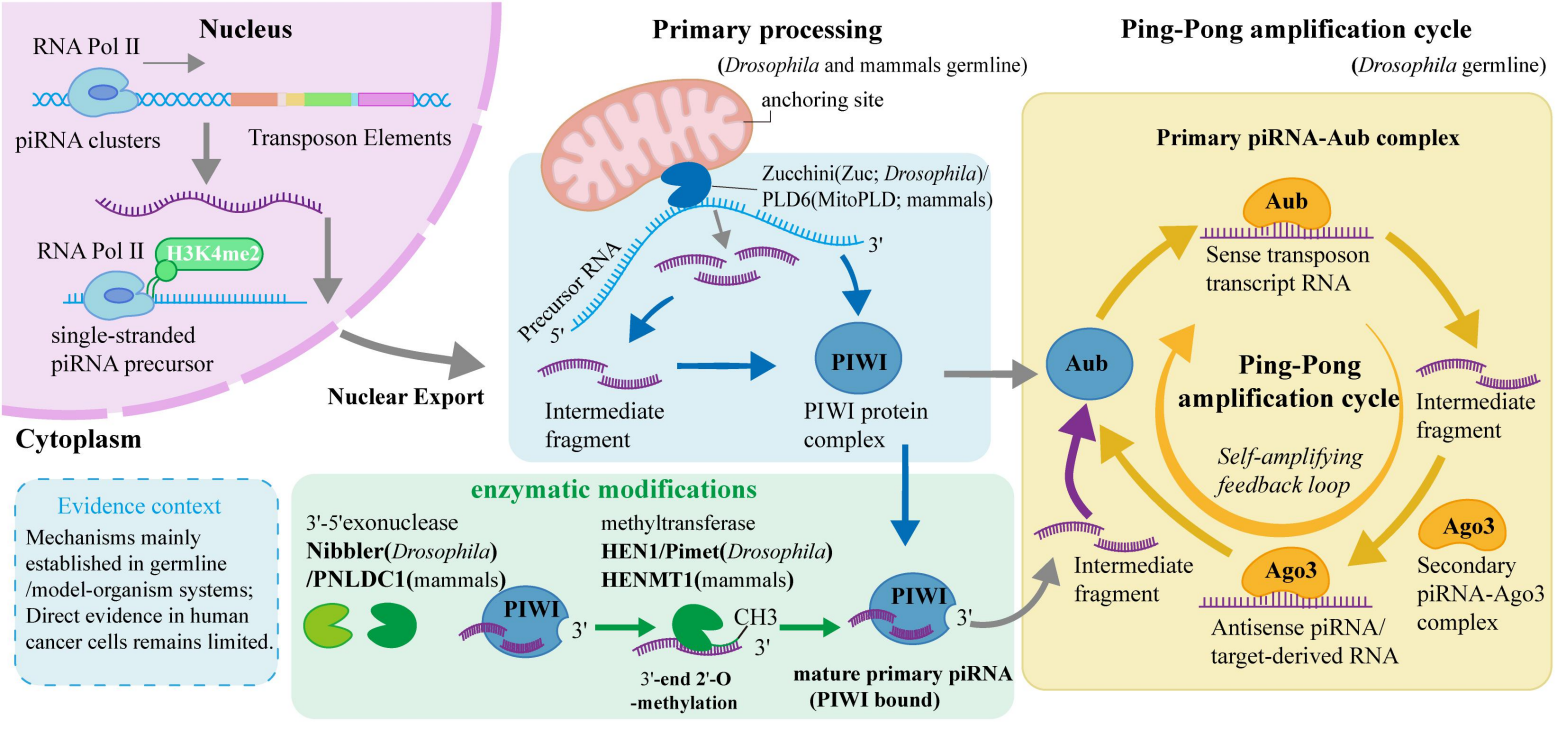
piRNA biogenesis: coordinated primary processing and ping-pong amplification. in the nucleus, piRNA clusters are transcribed by RNA polymerase ii to produce precursor RNAs that are exported to the cytoplasm. During primary processing, precursor RNAs are cleaved by zucchini (fruit fly) or PLD6/MitoPLD (mammals), loaded into PIWI proteins, trimmed by nibbler (fruit fly) or PNLDC1 (mammals), and methylated at the 3′ end by HEN1/Pimet (fruit fly) or HENMT1 (mammals) to generate mature primary piRNAs. in the fruit fly germline, Aub and Ago3 participate in ping-pong amplification through reciprocal cleavage of transposon-derived RNAs. These mechanisms are established mainly in germline/model-organism systems, and direct evidence in human cancer cells remains limited.

Most steps described above were defined in animal germline models. Somatic and cancer-associated piRNA pathways may differ in PIWI composition and may lack a detectable ping-pong signature; therefore, individual components should be verified directly in each tumor context [4,16,17].

## The link between piRNAs and cancer

### Cancer-associated expression and functions

piRNAs were originally identified in germ cells for their canonical role in suppressing TEs. However, they have been increasingly demonstrated to exert functional roles in somatic cells in recent years. Their dysregulation has been associated with altered transposon control, cancer stem cell phenotypes, therapeutic resistance, and tumor recurrence [16]. Accumulating evidence indicates that aberrant expression of piRNAs is associated with multiple human cancers, including colorectal, breast, prostate, hematologic, cervical, lung, and ovarian cancers [3,24,33–35]. Their dysregulation has been associated with tumorigenesis and cancer progression in selected cancer types, and individual piRNAs may serve as candidate biomarkers or therapeutic targets pending further validation [24,34]. Nevertheless, the underlying causes of aberrant piRNA expression in tumors and the precise molecular mechanisms governing their roles in tumorigenesis and cancer progression remain poorly understood, which warrants further intensive investigation by the research community.

Emerging evidence demonstrates that piRNAs are expressed in a tissue-specific manner across multiple human tissues and regulate key signaling pathways, such as the PI3K/AKT/mTOR pathway. Selected piRNAs have been reported to promote malignant phenotypes in the cancer models studied to date, including unlimited replication, resistance to apoptosis, invasion and metastasis, induction of angiogenesis, metabolic reprogramming, and DNA damage tolerance [3]. Furthermore, accumulating evidence indicates that aberrantly expressed piRNAs in various cancers can serve as novel biomarkers for cancer screening and diagnosis, as well as therapeutic targets in selected cancer types, although their broader clinical applicability remains to be established [25]. Garcia-Borja et al. elaborated that the piRNA-PIWI axis plays a critical role in maintaining the undifferentiated phenotype of CSCs, which is considered a major cause of tumor recurrence after treatment. Targeting this axis for biomarker development or therapeutic intervention holds promise for alleviating therapeutic resistance [16]. Weng et al. performed piRNA expression profiling in colorectal cancer (CRC) and adjacent normal tissues using small RNA sequencing, followed by independent validation in three independent cohorts of CRC patients [36]. They identified that piR-1245 is overexpressed in CRC, directly silences multiple tumor suppressor pathways, promotes cell survival, migration, and invasion, and is strongly associated with advanced metastasis and shorter overall survival [36]. Mai et al. also confirmed the oncogenic role of piR-54265 in CRC and found that piR-54265 is stably present and readily detectable in patient serum [37]. Further investigation into its potential as a blood-based biomarker for population screening and clinical application measured serum piR-54265 levels in four distinct groups of subjects. Compared with controls, only CRC patients exhibited significantly elevated serum piR-54265 levels. Notably, serum piR-54265 showed higher sensitivity than several conventional circulating CRC markers in that study, supporting its potential for CRC screening, early detection, and clinical surveillance [37].

A systematic review and meta-analysis explored the association between piRNA expression and prognosis in cancer patients. Analysis of eligible studies revealed that dysregulated piRNA expression correlates with survival outcomes in cancer patients, suggesting that various piRNAs may play roles in cancer survival. The review particularly highlighted the role of piRNA-823 in cancer, finding that its overexpression is significantly associated with overall survival in patients with CRC and renal cell carcinoma (RCC). These findings suggest that piRNA expression profiles, including piR-823 expression, may have prognostic relevance in selected cancer types. However, their performance as prognostic biomarkers requires independent validation, and the reported context-dependent functions of piR-823 caution against assuming uniform prognostic or biological significance across malignancies [38]. Importantly, the oncogenic functions of piRNAs are not limited to tumor cells themselves. Ou et al. discovered that in breast cancer patients receiving chemotherapy, chemotherapy-induced senescent neutrophils secrete large amounts of exosomal piRNA-17560 via the STAT3 signaling pathway [39]. This exosomal piRNA upregulates the expression of fat mass and obesity-associated protein (FTO) in breast cancer cells, reduces m6A RNA methylation, and thereby enhances the stability and expression of ZEB1 transcripts. This induces epithelial-mesenchymal transition (EMT) and increases chemoresistance, indicating that senescent neutrophil-derived exosomes may serve as potential therapeutic targets for breast cancer and revealing the critical role of exosomal piRNAs in the tumor microenvironment [39].

Sex-specific piRNA expression has also been reported. Human fetal ovary and adult testis show different piRNA expression patterns, although developmental stage and tissue context must be considered [40]. Whether comparable sex-dependent regulation contributes to piRNA dysregulation in human cancers remains unclear and warrants sex-stratified investigation.

Taken together, these findings suggest that the relationship between piRNAs and epigenetic regulation is context-dependent rather than uniformly conserved across biological systems. Although mechanistic studies in model organisms provide a valuable framework for understanding PIWI-associated chromatin regulation, the extent to which these mechanisms operate in human cancer cells requires direct investigation. This distinction is particularly important when interpreting cancer-associated piRNA expression, because an observed association does not necessarily establish a causal role in tumor development. Studies in selected cancer types suggest that individual piRNAs may be associated with tumor-related phenotypes and, in some experimental models, may contribute functionally to cancer progression or treatment resistance. Certain circulating or exosomal piRNAs have also been investigated as candidate biomarkers, while piRNA-associated pathways have been proposed as potential therapeutic targets. However, the reproducibility, cancer specificity, and clinical utility of these findings require further validation. The interpretation of these associations is also constrained by challenges in somatic piRNA annotation and the need to distinguish altered expression from experimentally supported, PIWI-dependent mechanisms. Consequently, the diagnostic potential of specific piRNA signatures remains to be established through independent clinical validation.

### Reliability of piRNA identification in somatic and cancer tissues

An important limitation in cancer piRNA research is that sequence annotation alone does not establish a PIWI-associated piRNA. Some sequences annotated as piRNAs overlap with fragments derived from tRNAs, rRNAs, snoRNAs, YRNAs, snRNAs, or miRNA precursors, and these fragments can contribute substantially to small-RNA signals in somatic non-gonadal tissues [4,17]. Therefore, small-RNA sequencing and database matching should be supported, when possible, by additional evidence such as appropriate size and end chemistry, expression of the relevant PIWI protein, PIWI-RNA association, and functional perturbation. Differences among reported tumor piRNA profiles may therefore reflect sample type, sequencing protocols, annotation databases, or PIWI expression as well as biological variation.

### Distinguishing association from mechanistic evidence

Cancer-associated piRNA studies differ considerably in evidence strength. Altered expression or clinical association alone does not establish a causal role. Functional perturbation provides stronger support, whereas demonstration of PIWI association, direct interaction with an epigenetic or epitranscriptomic regulator, and rescue experiments provide progressively stronger mechanistic evidence. These distinctions are considered throughout the cancer-specific discussion and Table 1.

**Table 1.** Cancer-associated piRNAs with reported epigenetic or epitranscriptomic mechanisms.

piRNA	Cancer type	Expression	Mechanism / key factors	Evidence*	Ref.
piR-31470	Prostate cancer	Up	DNA methylation: PIWIL4-GSTP1; DNMT1/DNMT3A/MBD2	E/F/P/D	[41]
piRNA-823	Multiple myeloma	Up	DNA methylation: DNMT3A/DNMT3B; p16INK4A; VEGF	E/F	[42]
pi-sno75	Breast cancer	Down	Histone methylation/demethylation: PIWIL1/4-WDR5-MLL3/UTX; TRAIL	E/F/P/D	[43]
piR-27222	PM2.5-related lung cancer model	Up	m6A on target mRNA: eIF4B-WTAP; Casp8	E/F/D	[44]
piRNA-14633	Cervical cancer	Up	m6A on target mRNA: METTL14; CYP1B1	E/F/D/R	[45]
piRNA-30473	DLBCL	Up	m6A on target mRNA: WTAP; HK2	E/F/D/R	[46]
piR-26441	Ovarian cancer	Down	m6A on target mRNA: YTHDC1; TSFM	E/F/D/R	[47]

*Evidence codes: E, expression or clinical association; F, functional perturbation; P, PIWI association; D, direct molecular interaction; R, rescue or pathway-dependency/epistasis. Evidence codes summarize experiments reported in the cited study and do not imply independent cross-study validation.

## Epigenetic regulation of piRNA and their bidirectional crosstalk

piRNAs are predominantly expressed in germ cells, with low expression in somatic cells, but are aberrantly expressed in tumor cells. Recent studies suggest that dysregulated piRNA expression is closely associated with oncogenic factors and epigenetic modifications. piRNAs have been implicated in cancer-associated epigenetic regulation, primarily through studies examining how selected piRNAs influence DNA methylation, histone-associated regulation, or RNA-modification pathways in specific tumor models. By contrast, evidence that cancer-associated epigenetic alterations directly affect piRNA transcription or biogenesis and thereby contribute to tumor phenotypes remains comparatively limited [24]. In the following sections, we systematically analyze the effects of altered epigenetic modifications on piRNAs in both normal and tumor cells, to further elucidate their relationship with cancer development. Because most detailed mechanisms in Epigenetic regulation of piRNA biogenesis in physiological models were established in *D. melanogaster*, *M. musculus*, *Caenorhabditis elegans*, or other physiological models, species and experimental context are specified and these mechanisms are not treated as direct evidence for human cancer. This section therefore distinguishes mechanisms established in physiological models from findings demonstrated in cancer and from proposed regulatory relationships that require further validation.

### Epigenetic regulation of piRNA biogenesis in physiological models

#### DNA methylation in physiological models

The piRNA pathway safeguards the germline genome from transposon invasion and directs transposon methylation during germ cell development, shedding light on the role of piRNAs in the epigenetics of the germline genome [48]. DNA methylation and piRNAs act synergistically to form a multi-layered dynamic network, constituting a reciprocal remodeling process in which DNA methylation shapes piRNA biogenesis and is in turn reshaped by piRNAs.

Researchers analyzed piRNA expression and DNA methylation profiles of long interspersed nuclear elements (LINE1) and intracisternal A particle (IAP) retrotransposons in the testes of MILI and MIWI2 wild-type mouse fetuses. MILI (PIWIL2) participates in precursor piRNA processing, while MIWI2 (PIWIL4) localizes piRNAs to the nucleus to indirectly induce DNA methylation. DNMT1 primarily functions in maintaining methylation, whereas DNMT3A and DNMT3C contribute to de novo methylation, with DNMT3L acting as a cofactor [15,20,49]. Watanabe et al. discovered that MIWI2 specifically interacts with RNAs from piRNA-mediated DNA methylation regions in the piRNA loci, indicating that MIWI2 influences chromatin state through base pairing between piRNAs and nascent transcripts, thereby determining the target specificity of DNA methylation [50].

During mammalian germline development, DNA methylation is extensively erased in primordial germ cells and subsequently re-established in fetal male germ cells; piRNA-directed DNA methylation contributes to transposon remethylation during this latter phase [20,48]. In 2020, Zoch et al. identified SPOCD1 (SPOC domain-containing protein 1) as a key effector of piRNA-directed de novo DNA methylation, the study confirmed that SPOCD1 interacts with MIWI2 to mediate de novo DNA methylation, serving as a critical determinant of TE silencing [20]. In 2024, Zoch et al. identified loss-of-function variants in human SPOCD1 associated with male infertility and defective transposon silencing; analysis of these alleles led to identification of C19ORF84 [49]. In mouse fetal germ cells, C19ORF84 is required for the association of SPOCD1 with DNMT3L and links SPOCD1 to the de novo DNA-methylation machinery [49]. Additional studies have identified testis expressed 15 (TEX15) as a regulator of TE silencing, and both TEX15 and SPOCD1 have been demonstrated to be essential for piRNA-directed transposon methylation [49,51].

piRNA biogenesis requires RNA helicases to unwind the secondary structures of precursor RNAs, thereby facilitating the loading of PIWI proteins. Mouse VASA homolog (MVH) is a well-characterized RNA helicase essential for piRNA production. Consistently, MVH-deficient male germ cells exhibit reduced piRNA expression levels and significantly compromised DNA methylation, demonstrating that MVH plays a critical role in de novo DNA methylation and retrotransposon silencing [52]. These findings highlight the dynamic regulatory interplay between piRNA biogenesis and DNA methylation. Promoter hypermethylation of the piRNA pathway genes PIWIL1, PIWIL2, and Tudor domain containing 1 (TDRD1) has also been reported in bovine hybrid male sterility and was associated with diminished pachytene piRNA production [53].

Notably, 5hmC has emerged as a novel stable epigenetic modification that regulates mammalian spermatogenesis. Gan et al. analyzed the genome-wide distribution of 5hmC in eight distinct types of germ cells during mouse spermatogenesis [54]. They found that 5hmC is mapped to genomic regions associated with the expression regulation of repeat-related elements in piRNA precursor genes. The enrichment levels of 5hmC in these regions correlated with the expression levels of the corresponding piRNAs and underwent dynamic changes during spermatogenesis, suggesting that 5hmC is a potential key regulator of spermatogenic cell differentiation in the mouse genome [54].

Therefore, piRNA-mediated DNA methylation ensures transposon silencing, and DNA methylation in turn modulates the expression of piRNA genes to regulate piRNA abundance. The synergistic interplay among Morc1/SetDB1, the MILI‑MIWI2‑SPOCD1‑C19ORF84‑TEX15 axis, the DNMT3A‑DNMT3L complex, chromatin remodelers, histone modifications, and the piRNA pathway drives de novo DNA methylation and repression of TEs in the mammalian male germline. piRNAs can serve as molecular guides to recruit histone modifiers and chromatin remodeling proteins to genomic target loci, thereby facilitating de novo DNA methylation [55,56]. These detailed methylation mechanisms are established mainly in mammalian germline models; their direct operation in human cancer remains incompletely defined.

#### Histone modifications in physiological models

##### Histone methylation

Histone methylation and piRNA biogenesis form a bidirectional epigenetic-transcriptional feedback loop. On one hand, repressive histone methylation marks (H3K9me3) and activating marks (H3K4me2) dictate the chromatin environment of piRNA clusters, thereby regulating piRNA production. On the other hand, mature piRNA-PIWI complexes exert a reciprocal effect on chromatin by remodeling histone methylation patterns, which further promotes transposon silencing [57].

The histone H3 lysine 9 trimethylation (H3K9me3) mark is the transcriptional foundation of piRNA clusters. In the female germline of *D. melanogaster*, dual-strand piRNA clusters are transcribed from heterochromatic regions in a manner dependent on the H3K9me3 mark. Akkouche et al. demonstrated that transient Piwi depletion in *D. melanogaster* embryos leads to reduced H3K9me3 at piRNA clusters in the ovaries, accompanied by impaired ovarian piRNA biogenesis, accumulation of transposon transcripts, and female sterility, in contrast, Piwi depletion in adulthood does not disrupt piRNA clusters, indicating that piRNAs are epigenetically acquired in a Piwi-dependent manner during embryonic development [58]. These findings indicate that embryonic Piwi is required for establishing the H3K9me3-rich chromatin state that licenses piRNA clusters and permits subsequent Rhino occupancy [58]. Subsequent studies have shown that Rhino directly binds H3K9me3 via its chromodomain and recruits the piRNA biogenesis factor Cutoff to piRNA clusters, ensuring the transcription of piRNA precursors and licensing entry into the piRNA processing pathway [59].

Erasure of H3K4me2 is a critical prerequisite for piRNA-directed de novo DNA methylation. During mammalian male germ cell development, retrotransposon silencing proceeds in two sequential phases. In the initial piRNA-independent phase, promoter regions are enriched with the activating histone mark H3K4me2, and de novo DNA methylation has not yet been established. This is followed by the piRNA-dependent phase, where the PIWIL4 (MIWI2)-piRNA complex recruits the H3K4 demethylases KDM1A and KDM5B to erase H3K4me2. Subsequently, SPOCD1 and C19ORF84 connect MIWI2 to the de novo DNA methylation machinery, including DNMT3C, in fetal gonocytes [20,49]. Loss of PIWIL4 or these demethylases results in persistent H3K4me2, failure to establish de novo DNA methylation, transposon activation, and genomic instability. Thus, Nagamori et al. uncovered the mechanistic link between PIWIL4-mediated H3K4me2 demethylation and piRNA-dependent de novo DNA methylation, which is essential for robust transposon silencing [20,49,60].

Dysregulation of H3K36 methylation triggers a transposon-piRNA cascade. Lindehell et al. found that loss of histone H3 lysine 36 (H3K36) methylation, mediated by the methyltransferases Set2 and NSD, leads to increased transposon transcription in *D. melanogaster* somatic cells, this activates key genes in the piRNA pathway and preferential RNA production from dual-strand piRNA clusters [61]. Its relevance to tumor progression remains untested.

Current studies have established that H3K9me3 dictates the transcription of piRNA clusters, erasure of H3K4me2 is a prerequisite for piRNA-mediated de novo DNA methylation, and H3K36 methylation maintains the homeostatic balance between transposons and piRNAs by restricting their transcription. However, several critical questions remain to be actively addressed, including the species-specificity of the aforementioned histone modifications and the precise mechanism by which H3K36-piRNA dysregulation drives tumorigenesis in somatic cells. Addressing these questions will not only deepen our understanding of the epigenetic-transposon-piRNA regulatory axis but also provide novel therapeutic insights for cancer intervention.

##### Histone acetylation and phosphorylation

Histone acetylation and phosphorylation function independently to regulate piRNA biogenesis and transposon silencing. Tsai et al. reported that in the *D. melanogaster* germline, the acetyltransferase Enok promotes Rhino expression by acetylating H3K23, and directly enhances piRNA-cluster transcription by facilitating Rhino recruitment, loss of Enok results in the disappearance of the H3K23 acetylation mark, impaired Rhino loading, reduced piRNA abundance, and transposon activation [62]. In *C. elegans*, Zhang et al. found that Casein Kinase II (CK2) promotes piRNA production by directly phosphorylating the USTC complex component TOFU-4 [63]. Phosphorylation of TOFU-4 by CK2 facilitates USTC assembly and piRNA transcription. During aging, decreased CK2 activity leads to USTC disassembly, reduced piRNA production, and defects in piRNA-mediated gene silencing. Thus, CK2 is essential for the generation or stabilization of piRNA precursors [63]. Although studies on these two modifications remain relatively limited, these cross-species investigations at multiple regulatory levels provide novel insights into the dynamic regulation of the piRNA pathway. These mechanisms were established in *D. melanogaster* and *C. elegans* and should not be assumed to operate identically in human cancer.

##### Histone sumoylation

Sumoylation is a reversible post-translational modification (PTM) in which small ubiquitin-like modifier (SUMO) molecules are covalently attached to lysine residues of target proteins via the catalytic action of a series of enzymes. Zhu et al. found that piRNA genes in *C. elegans* undergo spatial clustering and phase-separate into liquid droplets, which recruit the piRNA upstream sequence transcription complex (UST) and the H3K27me3 reader UAD-2 [64]. The phase separation property and sumoylation status of UAD-2 form a complex regulatory network that coordinates piRNA transcription and biogenesis. GEI-17-mediated sumoylation by the SUMO E3 ligase disperses these liquid droplets and inhibits piRNA transcription, whereas the SUMO protease TOFU-3 promotes condensation and enhances piRNA transcription. Spatial clustering, phase separation, and desumoylation of piRNA loci collectively facilitate proper piRNA transcription [64]. In *D. melanogaster*, Andreev et al. revealed that Panoramix undergoes Piwi-dependent sumoylation on chromatin [65]. This modification is a prerequisite for the recruitment of the heterochromatic multi-zinc-finger protein Small ovary (Sov). The interaction between Panx and Sov proteins is essential for target silencing and heterochromatin formation, and it confines the crosstalk between the piRNA pathway and heterochromatin machinery exclusively to piRNA target loci [65]. In addition, Ninova et al. developed a proteomics-based strategy to profile the SUMOylome of *D. melanogaster* ovaries, demonstrating that sumoylation is widespread across proteins involved in heterochromatin regulation and multiple aspects of the transposon-silencing piRNA pathway [66]. The SUMO E3 ligase SU(Var)2–10 interacts with nuclear heterochromatin proteins of the piRNA pathway to assist SetDB1 in depositing the repressive epigenetic mark H3K9me3. It sumoylates key piRNA factors including Piwi, MAEL, Panx, and Spn-E, with only a small subset of proteins being transiently sumoylated. These sumoylation events play critical roles in piRNA-mediated transposon silencing in *D. melanogaster* [66,67]. Collectively, these findings demonstrate that sumoylation functions in a tightly regulated manner during piRNA biogenesis to ensure proper piRNA production and precise transposon silencing. The SUMO-dependent mechanisms described here are likewise model- and species-specific.

##### Histones and chromatin

piRNA biogenesis is primarily determined by the chromatin environment of their precursor transcripts. Recent studies have gradually revealed that histone modifications and chromatin remodeling collectively form an intricate interplay network that both dictates TE transcription and modulates piRNA processing efficiency, thus underscoring the importance of delineating the regulatory roles of histones and chromatin in piRNA biogenesis. Lwasaki et al. demonstrated that the Piwi-piRNA pathway in *D. melanogaster* transcriptionally silences specific targets through heterochromatin formation, and increases H3K9me3 and histone H1 deposition at these loci, which can induce progressive changes in nuclear architecture and chromatin state at transcriptionally silenced target loci [68]. Sienski et al. characterized CG9754/Silencio as an essential piRNA pathway factor required for Piwi-mediated transcriptional silencing in *D. melanogaster* [69]. CG9754/Silencio relays sequence information to the H3K9 methyltransferase SetDB1, which then enriches H3K9me3 and recruits HP1 to complete heterochromatinization and transcriptional silencing. Loss of CG9754/Silencio results in a drastic reduction in H3K9me3 levels at TEs genome-wide, indicating that CG9754/Silencio acts downstream of Piwi and upstream of SetDB1 as a core adaptor linking piRNA complexes to chromatin modifications [69]. However, H3K9me3 does not simply control transcription. Mohn described the complex composed of Rhino, Deadlock, and Cutoff (RDC) at dual-strand piRNA clusters in *D. melanogaster* ovaries. Rhino, a member of the HP1 family, is anchored to H3K9me3-marked chromatin via its chromodomain. SETDB1 catalyzes the H3K9me, a major heterochromatin mark, suggesting a functional link between heterochromatin and piRNA cluster biology [70]. A specific zinc-finger protein, Kipferl, specifically binds the H3K9me3 chromatin mark and acts as a critical cofactor for Rhino, recruiting Rhino to specific loci and stabilizing it on chromatin to promote transcription of dual-strand piRNA clusters. In Kipferl mutant *D. melanogaster*, Rhino is lost from most target chromatin loci, resulting in piRNA depletion and transposon derepression [71]. Casier et al. revealed that the histone demethylase Kdm3 erases H3K9me3 to prevent excessive transcription of BX2 (a piRNA-producing locus) and other dual-strand piRNA clusters [72]. This indicates that piRNA clusters require an optimal level of H3K9me3: excessive H3K9me3 leads to heterochromatinization of the clusters themselves and transcriptional repression, while insufficient H3K9me3 abrogates Rhino anchoring. The histone demethylase Kdm3 maintains this dynamic balance. A chromodomain-containing protein mediates heterochromatin-directed piRNA expression. Huang et al. showed that in *C. elegans*, piRNA clusters are enriched for H3K27me3 [73]. UAD-2, a putative reader of H3K27me3, recruits the upstream sequence transcription complex (USTC) to piRNA promoters. Transcription of piRNA clusters depends on the chromatin environment and the chromodomain-containing protein UAD-2, which regulates piRNA production by depositing the UAD-2-USTC complex. Loss of histone H3 lysine 27 methylation or UAD-2 results in reduced piRNA production [73]. As previously demonstrated by Zhu et al., sumoylation of UAD-2 by the *C. elegans* SUMO E3 ligase GEI-17 drives phase separation, while desumoylation by TOFU-3 promotes condensation, thereby regulating piRNA transcription [64].

piRNA clusters in *D. melanogaster* possess a unique chromatin structure. Iwasaki et al. found that Piwi interacts with linker histone H1, and Piwi depletion reduces H1 density at a subset of TEs, leading to their derepression, this demonstrates that Piwi-piRISC regulates the interaction between chromatin components and target loci, maintaining TE silencing by modulating chromatin accessibility [74]. Thomas et al. showed that Piwi recognizes cognate targets complementary to piRNAs and induces transcriptional repression upon correct target recognition by establishing a repressive chromatin state [75]. Onishi et al. proposed that Piwi rapidly represses the transcription of Brahma transposons in ovarian somatic cells via Maelstrom, promoting heterochromatin formation and transposon silencing [76]. These findings suggest that these pathways coordinately regulate chromatin organization to mediate genome-wide piRNA targeting of TEs [74]. Klenov et al. described that nuclear Piwi depletion results in a significant increase in H3K4me2 levels, while H3K9me3 and HP1a levels remain unchanged [77]. They found that most transposons enriched with H3K9me3/HP1 are resistant to Piwi loss, indicating that Piwi contributes to transposon silencing in the ovary through heterochromatin pathways independent of H3K9me3 [77]. Subsequently, Iwasaki et al. pointed out that chromatin-associated lamins are reduced following Piwi depletion, Piwi-piRNA-mediated TE regulation is initiated by altering nuclear localization and removing active histone marks, and these progressive changes further lead to the formation of stable heterochromatin structures [68]. This establishes that Piwi-piRNA-mediated target TE regulation is linked to nuclear localization, histone modifications, and chromatin remodeling. Histones and chromatin are active regulators of piRNA biogenesis. Most mechanisms in this subsection derive from *D. melanogaster* models; direct human cancer evidence is more limited and is discussed in piRNA-mediated DNA methylation in cancer.

#### RNA modifications in physiological models

m6A modification is catalyzed by methyltransferases, demethylases, and m6A-binding proteins. m6A modification can contribute to TE silencing [78]. In mouse embryonic stem cells, METTL3/YTHDC1-dependent m6A of retrotransposon RNAs supports SETDB1/TRIM28-associated heterochromatin independently of the piRNA pathway [79]. In the male mouse germline, SPOCD1 separately acts downstream of MIWI2 in piRNA-directed DNA methylation [20]. Kojima-Kita et al. designed a fusion protein containing MIWI2 and a zinc finger (ZF) domain that recognizes the promoter region of type A LINE1 genes to induce DNA methylation, even in the absence of piRNAs, this fusion protein can be recruited to LINE1 loci and function as an effector of de novo DNA methylation [80]. These findings suggest that RNA modifications and piRNA functions can act either synergistically or independently (Figure 3). These studies mainly concern retrotransposon or other RNA substrates; the best-established covalent modification of mature piRNAs remains 3’terminal 2’-O-methylation. The SPOCD1-related evidence here is primarily from mouse germline models, whereas the human cancer studies in piRNA-m6A crosstalk in cancer mainly concern m6A on target mRNAs.

**Figure 3.**
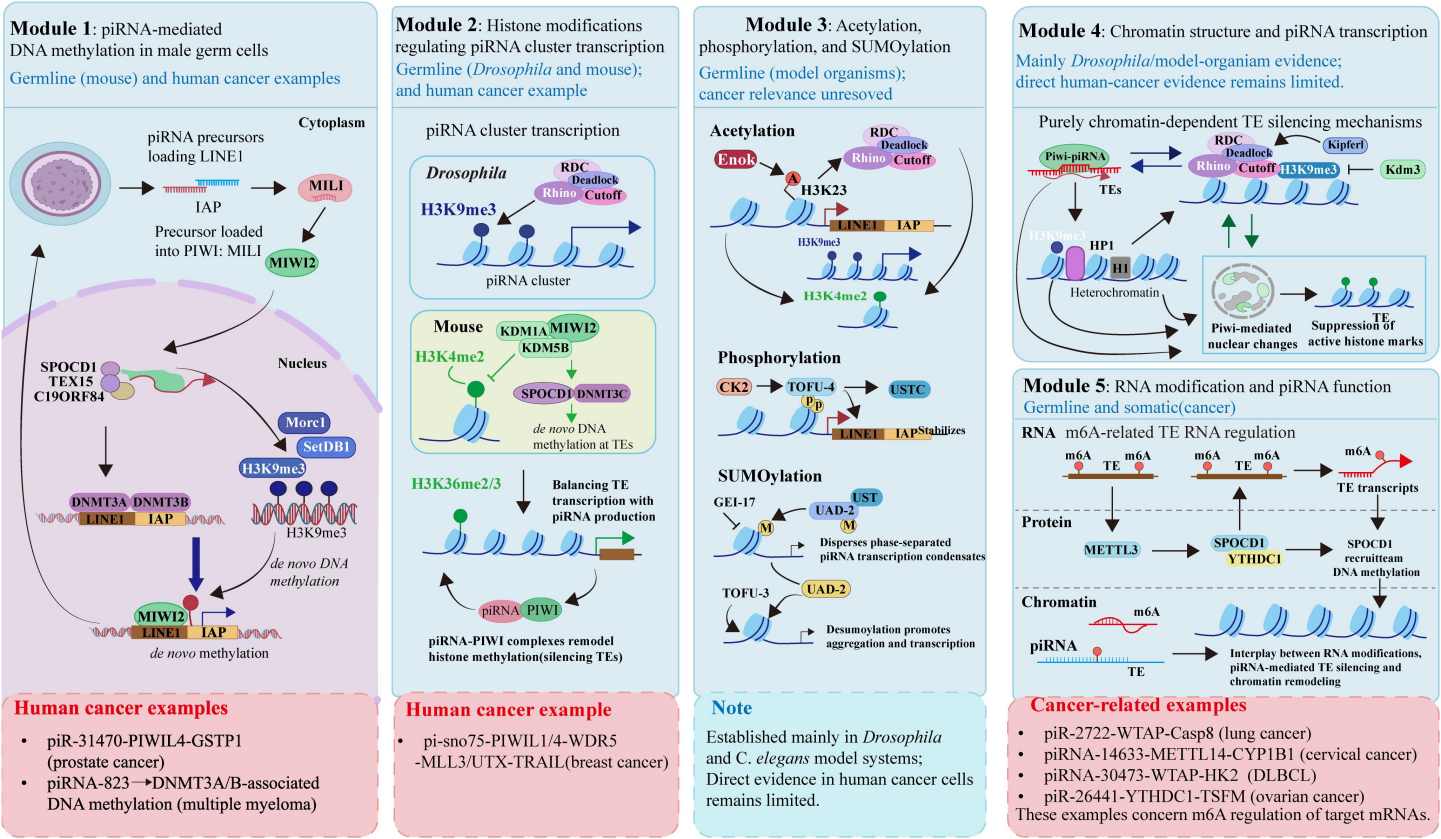
Epigenetic and epitranscriptomic mechanisms of piRNAs in germline and cancer. **Module 1**: piRNA-mediated DNA methylation in the mouse male germline, with selected human cancer examples. **Module 2**: histone-modification-dependent regulation of piRNA cluster transcription in fruit fly and mouse, with a representative human cancer example. **Module 3**: histone acetylation, phosphorylation, and SUMOylation in piRNA-related transcriptional regulation; these mechanisms are established mainly in model organisms, and direct evidence in human cancer cells remains limited. **Module 4**: chromatin structure and nuclear architecture in piRNA transcription and transposon repression; evidence is mainly from fruit fly and other model systems, with limited direct evidence in human cancer. **Module 5**: RNA modification and piRNA function, including m6A-related regulation in the piRNA pathway and selected cancer-associated examples involving m6A regulation of target mRNAs. Across modules, mechanisms established in germline/model-organism systems are distinguished from cancer-related evidence.

### Bidirectional regulatory axis of epigenetics and piRNAs in cancer cells

#### Epigenetic regulation of piRNA expression in cancer

In physiological germline models, epigenetic modifications regulate piRNA-cluster transcription and biogenesis. In contrast, cancer-specific evidence that epigenetic alterations cause changes in piRNA expression or biogenesis that subsequently contribute to tumor phenotypes remains limited. Most cancer studies have instead examined how dysregulated piRNAs influence DNA methylation, histone-associated regulation, or RNA-modification pathways. Accordingly, the following sections distinguish established mechanisms from cancer-specific associations and hypotheses, with representative findings summarized in Table 1.

The available evidence supports an important asymmetry in our current understanding of the epigenetic-piRNA relationship in cancer. In several cancer models, individual piRNAs have been linked to changes in DNA methylation, histone-associated regulation, or m6A-related pathways. However, the reverse sequence-cancer-associated epigenetic alteration leading to altered piRNA expression or biogenesis, followed by a demonstrable change in tumor phenotype-has not been established with comparable consistency. This asymmetry is therefore framed here as a major knowledge gap rather than as evidence for an established bidirectional mechanism in cancer. Mechanisms identified in germline systems provide plausible hypotheses for how DNA methylation, chromatin accessibility, histone modifications, or RNA-processing factors might influence piRNA production. Nevertheless, these mechanisms should not be directly extrapolated to human tumors without cancer-specific validation. In particular, an association between an epigenetic mark and altered piRNA abundance does not establish that the epigenetic change is upstream of the piRNA alteration, nor that the latter mediates a malignant phenotype.

Future studies should therefore test the proposed regulatory sequence explicitly. This will require locus-specific manipulation of the relevant epigenetic mark, measurement of precursor and mature piRNA abundance, confirmation of PIWI association where appropriate, and functional experiments demonstrating whether the resulting piRNA change contributes to the cancer phenotype. Rescue experiments would provide additional evidence of mediation. Until such evidence is available, the proposed epigenetic alteration-piRNA dysregulation-cancer phenotype pathway should be regarded as a compelling but incompletely validated model rather than a generally established mechanism in human cancer.

#### piRNA-mediated DNA methylation in cancer

Accumulating evidence indicates that DNA methylation patterns in malignant tumor cells exhibit marked differences from those in normal cells [81]. Emerging studies have demonstrated that piRNAs participate in the epigenetic regulation of cancer and are emerging as key players in this process. Zhang et al. found that piR-31470 is highly expressed in prostate cancer and forms the PIWIL4/piR-31470 complex by binding to PIWIL4 [41]. This complex binds to nascent RNA transcripts of GSTP1 and recruits DNA methyltransferase DNMT1, DNMT3A, and methyl-CpG binding domain protein 2 (MBD2). It initiates and maintains hypermethylation and silencing of GSTP1, thereby promoting prostate carcinogenesis [41]. Yan’s team confirmed that piRNA-823 is upregulated in multiple myeloma (MM). Inhibition of piRNA-823 reduces DNMT3A and DNMT3B expression and global DNA methylation, re-expresses the methylation-silenced tumor suppressor p16INK4A, and decreases VEGF-associated proangiogenic activity, supporting a functional role for piRNA-823 in de novo DNA methylation in this MM model [42]. Collectively, piRNAs can influence DNMT-dependent methylation pathways in defined tumor models. This regulatory axis may provide candidate targets for further mechanistic investigation in cancer. These findings provide mechanistic examples in defined cancer models but should not be generalized to all tumor types.

#### piRNA-mediated histone modifications in cancer

He et al. first demonstrated that a piRNA derived from the tumor-suppressive lncRNA Growth Arrest Specific 5 (GAS5), designated pi-sno75, binds to PIWIL1/4 proteins and interacts with WDR5 [43]. This complex recruits the hCOMPASS-like complex containing MLL3 and UTX, which upregulates the transcription of Tumor Necrosis Factor-related apoptosis-inducing ligand (TRAIL) by inducing H3K4 methylation and H3K27 demethylation, thereby triggering tumor cell apoptosis and suppressing breast cancer cell growth [43]. This study provides a canonical paradigm for the piRNA-histone methylation-cancer regulatory axis. However, the mechanisms by which other histone modifications including acetylation and phosphorylation regulate piRNAs and contribute to their dysregulation in tumors remain largely understudied. Future investigations should elucidate the effects of histone acetyltransferases and other epigenetic enzymes on piRNA biogenesis and function in cancer cells, which may facilitate the discovery of novel combined intervention strategies targeting both epigenetic modifications and the piRNA pathway. This represents a locus-specific mechanism in breast cancer models; comparable histone-centered piRNA mechanisms remain less well characterized in other tumors.

#### piRNA-m6A crosstalk in cancer

m6A is one of the most abundant internal RNA modifications. Recent cancer studies indicate that piRNAs can modulate m6A-related pathways and thereby affect target-mRNA stability or expression [44–47]. Ma et al. analyzed piRNA profile alterations in PM2.5-induced lung cancer models and found that piR-27222 is highly expressed and inhibits cell death in an m6A-dependent manner, piR-27222 directly binds eIF4B, promotes the protein levels of the m6A methyltransferase WTAP through deubiquitination and stabilization of EIF4B, and increases m6A modification in Casp8 transcripts, thus conferring resistance to PANoptosis in lung cancer cells [44]. Xie et al. discovered that piRNA-14633 is upregulated in cervical cancer and specifically interacts with METTL14, with CYP1B1 being the key downstream target of METTL14 [45]. Increased m6A modification promotes the proliferation, migration, and invasion of cervical cancer cells. This study revealed that piRNA-14633 upregulates CYP1B1 expression via METTL14-mediated m6A methylation, thereby driving malignant transformation of cervical cancer cells [45]. In diffuse large B-cell lymphoma (DLBCL), Han’s team showed that piRNA-30473 is highly expressed and exerts oncogenic effects by upregulating WTAP to increase m6A levels of its key target gene HK2. Thus, the piRNA-30473/WTAP/HK2 axis participates in tumorigenesis by modulating m6A modification in DLBCL, suggesting potential prognostic relevance and possible therapeutic value in the cancer type studied [46]. Yuan et al. found that piR-26441 is aberrantly downregulated in ovarian cancer, piR-26441 directly binds to and increases YTHDC1, promotes m6A-dependent destabilization of TSFM mRNA, reduces mitochondrial oxidative phosphorylation, and suppresses ovarian cancer progression, thus, loss of piR-26441 may weaken the YTHDC1/TSFM tumor-suppressive pathway [47]. In these cancer studies, the m6A-modified substrates are primarily target mRNAs rather than the mature piRNAs themselves.

Collectively, cancer-associated piRNAs can regulate m6A writers or readers and thereby alter target-mRNA stability or expression. Conversely, whether m6A directly modulates piRNA precursor stability or mature piRNA biogenesis in cancer remains insufficiently established. Therefore, further clarification of the piRNA-m6A interaction axis may improve mechanistic understanding and help evaluate its potential biomarker or therapeutic relevance (Table 1). Table 1 summarizes these cancer-associated piRNAs and distinguishes expression, functional, PIWI-association, direct-interaction, and rescue/pathway-dependency evidence.

An illustrative example of the context-dependent and potentially divergent roles reported for candidate piRNAs is piR-823, which has been associated with distinct biological effects across malignancies. In gastric cancer, piR-823 was reported to be downregulated, and restoration of its expression suppressed tumor cell growth in vitro and in xenograft models, supporting a tumor-suppressive role in this context [82]. In contrast, piR-823 was found to be upregulated in colorectal cancer (CRC), where its inhibition reduced cell proliferation and promoted apoptosis. Mechanistic analyses further implicated HSF1 phosphorylation and the regulation of heat shock protein expression in these effects [83]. In multiple myeloma, piR-823 has also been associated with tumorigenesis through the regulation of de novo DNA methylation and angiogenesis [42]. These findings argue against assigning piR-823 a uniformly oncogenic or tumor-suppressive role across cancers. Instead, its biological effects may depend on the cellular context, the availability of interacting molecules, and the downstream pathways engaged in each malignancy.

Importantly, however, these studies do not constitute a direct comparison of piR-823 function under matched experimental conditions. Differences in cancer type, model system, and the molecular endpoints examined may contribute to the apparent divergence in reported effects. The strength of mechanistic evidence also varies: altered expression and associated clinical features are not equivalent to functional perturbation, and functional effects alone do not establish dependence on a canonical PIWI-piRNA pathway. Accordingly, interpretation of cancer-associated piRNAs should distinguish expression-level associations from experimentally supported functions and from mechanisms validated through specific interaction, pathway-dependency, and rescue experiments. Comparative studies using rigorous RNA-sequence validation and standardized functional assays will be important for determining whether reported differences reflect genuine context-dependent biology, methodological variation, or both.

## Future research directions and conclusions

### Therapeutic potential of the piRNA-epigenetic axis

Recent studies have revealed a close association between histone modifications and temozolomide (TMZ) resistance in gliomas. TMZ exerts potent inhibitory effects on glioma cell growth and induces cell cycle arrest; however, dysregulation of histone modifications mediates tumor resistance to this chemotherapeutic agent, making histone modification-related proteins potential therapeutic targets for glioma treatment [84]. Numerous epigenetic drugs have been developed to date, with a substantial number already being investigated for CRC treatment. Both preclinical studies and clinical trials have investigated whether these approaches can improve therapeutic outcomes [85]. A major limitation of epigenetic drugs in solid tumors is their poor selectivity and associated off-target effects, which may lead to adverse reactions due to non-specific targeting. Nevertheless, clinical activity of epigenetic agents has been more clearly established in hematological malignancies [86].

Direct therapeutic manipulation of piRNAs remains preclinical. Oncogenic piRNA pathways could in principle be inhibited and tumor-suppressive piRNAs restored. The PIWIL4/piR-31470-GSTP1 methylation axis provides a model for testing whether piR-31470 inhibition can reverse gene silencing [41]. However, delivery, PIWI dependence, and uncertain somatic piRNA annotation remain major barriers; no piRNA-directed epigenetic therapy is clinically established.

### Epigenetic drugs and dietary bioactives

Epigenetic diets have also been investigated for potential anticancer effects in various studies. Epigenetic diets are dietary patterns rich in dietary bioactives, polyphenols, and other secondary metabolites, which are primarily found in fruits, vegetables, spices, and other plant parts. These dietary bioactive compounds can influence epigenetic regulatory enzymes, including DNA methyltransferases (DNMTs) and histone deacetylases (HDACs) [86]. For example, sulforaphane (SFN), abundant in cruciferous vegetables, inhibits inflammatory markers in various cancer cells. It has been shown to suppress DNMT and HDAC activities, exerting diverse chemopreventive and therapeutic effects against multiple cancers. Curcumin inhibits cancer progression by altering cellular signaling pathways through epigenetic regulation, including modulating histone acetyltransferases (HATs), HDACs, and histone methyltransferases (HMTs), as well as inhibiting DNMTs and histone modifications. However, the systemic bioavailability of these bioactive compounds at tumor sites is relatively low, and current mechanistic and clinical evidence does not establish epigenetic diets as inherently safer or more effective than pharmacological epigenetic therapies.

DNMT inhibitors such as azacitidine and decitabine, and HDAC inhibitors such as vorinostat and romidepsin, represent clinically applicable examples of epigenetic therapies. Azacitidine/decitabine are used mainly in myeloid malignancies and vorinostat/romidepsin in cutaneous T-cell lymphoma [86], demonstrating clinical druggability but not piRNA-locus specificity. However, their limitations include incomplete specificity, off-target effects, and other adverse reactions.

### dCas9-based epigenome editing and future applications in piRNA research

Programmable dCas9-based epigenome editing provides a locus-specific strategy for investigating whether local chromatin states regulate transcription at candidate piRNA-producing loci. In this approach, dCas9 is fused to an epigenetic effector and guided to a selected genomic region, allowing targeted transcriptional activation through p300-mediated histone H3 lysine 27 acetylation, transcriptional repression through KRAB-associated repressive chromatin modifications, or targeted DNA methylation through DNMT3A [87–89]. Unlike conventional nuclease-based CRISPR/Cas9 genome editing, these approaches are designed to modify local epigenetic states without intentionally altering the underlying DNA sequence. Building on these established capabilities, dCas9-based activators or repressors could, in principle, be used to investigate whether changes in the chromatin state of candidate piRNA-producing loci alter piRNA precursor transcription and mature piRNA abundance. Subsequent analyses could examine whether these changes affect PIWI association, downstream target regulation, and cancer-associated phenotypes. Appropriate controls should include non-targeting guide RNAs, independent guide RNAs targeting the same locus, and effector-specific or catalytically inactive controls where applicable. Changes in candidate piRNA abundance should be assessed using small-RNA sequencing and orthogonal validation, followed by PIWI-association assays where appropriate and functional experiments, including rescue assays, to establish biological relevance.

Beyond their value for mechanistic validation, piRNA–PIWI pathways may eventually provide opportunities for targeted epigenetic modulation. In selected cancer models, piRNA-associated complexes have been linked to locus-specific epigenetic regulation, including the recruitment of DNA methylation machinery to the GSTP1 locus by the PIWIL4/piR-31470 complex in prostate cancer and the recruitment of MLL/COMPASS-like complexes by pi-sno75-associated machinery to regulate TRAIL transcription in breast cancer [41,43]. A related proof of concept comes from a study using an MIWI2–zinc-finger fusion to induce DNA methylation at LINE1 promoters in mouse embryonic male germ cells; however, this strategy can function independently of piRNAs and should not be interpreted as evidence for an established piRNA-guided editing platform [80]. These findings provide a rationale for investigating whether defined piRNA-associated mechanisms could be adapted for locus-directed regulation of gene expression. Nevertheless, the feasibility of engineering piRNA-guided epigenetic effects in human cancer cells remains to be demonstrated, particularly because PIWI expression, piRNA processing, target recognition, and downstream effector recruitment may differ between germline and somatic contexts.

The potential therapeutic relevance of these strategies therefore depends on several unresolved issues, including the reliability of somatic piRNA annotation, the reproducibility of piRNA–PIWI interactions, locus and cell-type specificity, delivery efficiency, and unintended epigenetic effects at other genomic regions. An integrated experimental framework combining locus-specific dCas9 perturbation, small-RNA profiling, PIWI-association assays, direct measurements of epigenetic marks, and functional rescue experiments could help distinguish whether an epigenetic alteration is an upstream cause of piRNA dysregulation or merely accompanies broader transcriptional changes. At present, the most immediate value of dCas9-based epigenome editing in piRNA research is likely to lie in the mechanistic validation of candidate piRNA-producing loci and their regulatory mechanisms, rather than established cancer therapy. Direct therapeutic manipulation of piRNAs remains an investigational strategy, and piRNA-directed epigenetic therapy has not been established as a clinical treatment.

### Challenges in measuring epigenetic marks

Measuring epigenetic changes during cancer development is complicated by intratumoral heterogeneity, changing cell composition, limited serial tissue, and the fact that DNA methylation, histone marks, RNA modifications, and piRNAs usually require different assays. Patient-matched longitudinal sampling and low-input or single-cell approaches can reduce these problems. scNMT-seq jointly profiles DNA methylation, chromatin accessibility, and transcription, whereas CUT&Tag profiles histone marks from small cell numbers [90,91]. Candidate piRNA pathways should then be confirmed in the same model using small-RNA sequencing, PIWI-association assays, and locus-specific validation.

### Conclusions

The PIWI-piRNA pathway has well-established roles in transposon silencing and epigenetic regulation in germline models. However, the extent to which these mechanisms are conserved in human somatic tissues and directly contribute to cancer development remains context-dependent. Studies in selected cancer types have linked individual piRNAs to DNA methylation, histone-associated regulation, and m6A-related pathways, but the strength of mechanistic evidence varies across studies, and these findings should not be generalized to all malignancies.

An important unresolved question is whether cancer-associated epigenetic alterations directly influence piRNA transcription or biogenesis and thereby contribute to tumor phenotypes. Although this regulatory direction is biologically plausible, the complete causal sequence remains insufficiently validated in human cancer. Reliable piRNA annotation, confirmation of PIWI association where appropriate, locus-specific epigenetic perturbation, and functional validation, including rescue experiments, will be essential to distinguish causal mechanisms from secondary molecular changes.

Future research should prioritize cancer-specific mechanistic validation and standardized assessment of piRNA expression and function. Although selected piRNAs may eventually have diagnostic, prognostic, or therapeutic relevance, their clinical utility requires independent validation, and direct piRNA manipulation remains investigational. The epigenetic–piRNA axis therefore represents a promising framework for mechanistic and translational research, rather than a clinically validated platform for cancer diagnosis or treatment.

## Data Availability

This article is a narrative literature review and did not generate or analyze new experimental datasets.
